# The Missed Opportunity: HIV, Hepatitis C Virus (HCV), and Hepatitis B Virus (HBV) Positive Patients in Neoadjuvant and Perioperative Immunotherapy Clinical Trials for Lung Cancer

**DOI:** 10.7759/cureus.51265

**Published:** 2023-12-29

**Authors:** Javier David Benitez Fuentes

**Affiliations:** 1 Medical Oncology, Velindre Cancer Centre, Cardiff, GBR; 2 Medical Oncology, Centro Integral Oncológico Clara Campal (CIOCC) Hospital Universitario HM Sanchinarro, Madrid, ESP

**Keywords:** neoadjuvant, global health, clinical trials, inclusivity, immunotherapy, bioethics and ethics in research, hbv, hcv, hiv, lung cancer

## Abstract

This editorial addresses a critical oversight in recent clinical trials on neoadjuvant or perioperative immunotherapy for lung cancer, the exclusion of patients with human immunodeficiency virus (HIV), hepatitis C virus (HCV), and hepatitis B virus (HBV). The ethical implications of this exclusion are highlighted, demonstrating how it undermines principles of inclusivity and equity in clinical research. We emphasize the necessity to include these patients to enhance the generalizability of trial findings. We suggest that trial eligibility criteria be revised, and collaborations with patient advocacy groups be initiated to ensure more inclusive future trials. This approach aims to uphold ethical research practices, yielding robust, representative data, and ultimately improving patient care in oncology.

## Editorial

The field of oncology has missed a significant opportunity in the recently published clinical trials on neoadjuvant and perioperative immunotherapy for lung cancer [1-4]. Various prominent studies failed to incorporate patients with human immunodeficiency virus (HIV), hepatitis C virus (HCV), and hepatitis B virus (HBV) infections [1-4] despite the guidance issued by the U.S. Food and Drug Administration (FDA) [5] and the National Comprehensive Cancer Network [6] advocating for their inclusion.

This oversight undermines the ethical principles of inclusivity and equity in clinical research but also hampers the generalizability of trial findings to the broader population of lung cancer patients. Excluding this population denies them access to potentially lifesaving therapies and deprives clinicians of much-needed critical evidence to guide treatment decisions. The exclusion of these individuals overlooks the higher incidence of cancer in this group compared to the general population [7-10]. Emerging data from real-world observations and certain focused studies suggest that immunotherapy can be safely and effectively administered to patients with HIV, HCV, and HBV, especially when their viral infections are well-managed [11-17]. This approach necessitates close collaboration with infectious disease specialists to ensure that the viral status is optimally managed. For HBV and HCV, this includes pre-treatment screening and regular monitoring. HIV patients should have a stable condition, as indicated by specific viral load and CD4+ T-cell counts, prior to starting immunotherapy [7]. Adjusting clinical trial eligibility criteria to include these patients when their infection is effectively managed could enhance the relevance and applicability of the trial outcomes, fostering a deeper understanding of the benefits and risks of these treatments in a more diverse patient population.

Future trials in immunotherapy for lung cancer should actively include patients with HIV, HCV, and HBV. This can be achieved by revising trial eligibility criteria and ensuring that trial designs accommodate the unique medical needs of these populations. Expanding eligibility criteria to include patients with chronic viral infections will enhance the validity of study results and the understanding of immune checkpoint inhibitors. Collaborating with patient advocacy groups and community healthcare providers can also help ensure the representation of these populations in clinical research.
